# High‐Brightness and Ultra‐Long‐Lifetime Spin Quantum Dot Light‐Emitting Diodes Enabled by Efficient Injection of Spin‐Polarized Holes Through a Hybrid Hole Transport Layer

**DOI:** 10.1002/advs.77899

**Published:** 2026-09-27

**Authors:** Fengqi Qiu, Hechun Zhang, Wenbo Liu, Guiming Zhong, Fumin Lu, Yuqi Wang, Fujin Ai, Kai Wang, Dan Wu

**Affiliations:** ^1^ Guangdong Provincial Key Laboratory of Low‐Altitude Intelligent Perception and Navigation Safety College of New Materials and New Energies Shenzhen Technology University Shenzhen China; ^2^ State Key Laboratory of Optical Fiber and Cable Manufacture Technology Department of Electronic and Electrical Engineering Southern University of Science and Technology Shenzhen China; ^3^ College of Health Science and Environmental Engineering Shenzhen Technology University Shenzhen China

**Keywords:** chiral perovskite, circularly polarized electroluminescence, colloidal quantum dots, hybrid hole transport layer, Spin light‐emitting diodes

## Abstract

Spin‐polarized quantum dot light‐emitting diodes (spin‐QLEDs) offer a package solution for advanced photonic applications, such as quantum information processing. However, the performance of these devices is threatened by integration of chiral layers due to greatly compromised interfacial hole transport and deteriorate film morphology. In this work, a hybrid hole transport layer (HTL) engineering strategy is proposed to simultaneously tackle the two key issues. On one hand, enhanced surface hydrophilicity of the HTL enables the growth of a high‐quality chiral perovskite layer as a spin filter. On the other hand, tri‐channel hole transport mechanism is first unraveled theoretically and experimentally to realize high‐mobility pathways, stepwise injection, and bypass the resistive limitations of traditional HTL. The resulting spin‐QLEDs demonstrated an ultralong *T*_50_ lifetimes at 100 cd/m^2^ to be 3968.9 h which is orders of magnitude higher than other reports. Moreover, the spin‐QLEDs exhibit a recorded luminance of 14,370 cd/m^2^ for red‐light‐emitting devices, alongside an external quantum efficiency of 6.68% and a circularly polarized electroluminescence asymmetry factor (*g*_CP‐EL_) of 0.026. This hybrid HTL architecture offers a reliable route toward long‐lived and high‐brightness spin‐optoelectronic devices.

## Graphical abstract

This work employs a hybrid hole transport layer to enhance hole transport efficiency, resulting in improved performance for the final device. It achieves an external quantum efficiency of 6.68%, a circularly polarized electroluminescence asymmetry factor of 0.026, and a high luminance of 14,370 cd/cm^2^, representing the highest luminance reported to date for red spin quantum dot light‐emitting diodes.

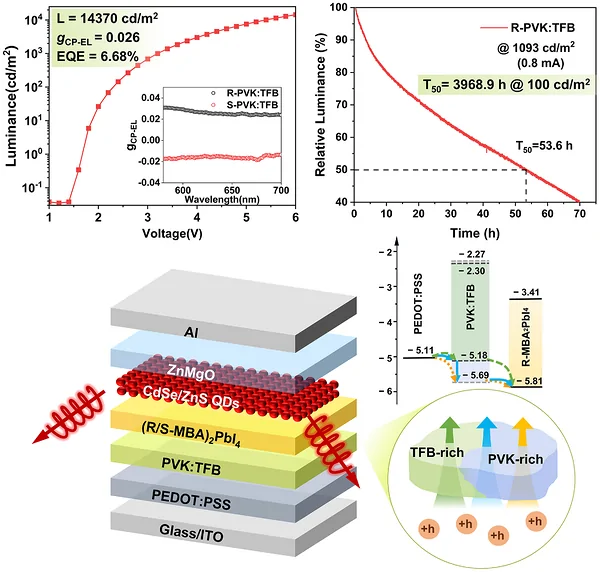

## 1 Introduction

Circularly polarized light (CPL) could be widely applied in next‐generation photonics, with potential in high‐density information storage [1], advanced biomedical imaging [2], secure encryption [3], and immersive three‐dimensional (3D) displays [4]. Traditionally, generating circularly polarized electroluminescence (CP‐EL) needs external linear polarizers and quarter‐wave plates which may impede device miniaturization. To circumvent this constraint, spin‐polarized light‐emitting diodes (spin‐LEDs) have emerged as a promising alternative, enabling the direct emission of CPL through the manipulation of spin‐polarized carrier generation and recombination under room temperature. The operational principle of these devices increasingly relies on the chiral‐induced spin selectivity (CISS) effect [5]. In these systems, the intrinsic lack of inversion symmetry creates a spin‐dependent potential to filter charge carriers based on their different spin angular momentum and therefore allows for the precise modulation of CP‐EL handedness (σ+ and σ−) [6].

Over the past half‐decade, the research of spin‐LEDs has seen a rapid expansion in chiral emitting materials, including perovskite nanocrystals, perovskite quantum dots (QDs) and II‐VI group colloidal QDs [7, 8, 9]. Bread et al. pioneered the integration of 2D chiral perovskites as spin‐filters with achiral perovskite nanocrystal emitters, achieving room‐temperature CPL with a polarization (*P*_CP‐EL_) of ±2.6% [10]. More recently, Song et al. (2025) developed multifunctional chiral perovskite QDs that act as both spin‐filters and radiative centers [11]. Despite these advances, II‐VI colloidal QDs remain uniquely attractive due to their tunable bandgaps, narrow emission linewidths, near‐unity quantum yields, and long working time compared to perovskite emitters [12]. Early efforts to harness these properties include chiral CdSe/CdS quantum rods and chiral metal‐organic frameworks (MOFs) paired with CdSe/ZnS QDs, the latter of which achieved a luminance of 1,000  cd/m^2^ [13, 14, 15]. Our group previously demonstrated that QD/2D‐chiral‐perovskite composites enhance both CPL activity and structural stability, yielding devices with an asymmetry factor (*g*_CP‐EL_) of 1.6 × 10^−2^ and a high luminance of 5,538 cd/m^2^ [16, 17, 18, 19]. However, dimming spin‐LEDs below 10000 cd/m^2^ can hardly be of any practical use which largely stems from the inferior interfacial quality at the hole transport layer (HTL) and the poor spin‐polarized carrier transport. A typical example of high‐mobility transport polymers as poly(9,9‐di‐n‐octylfluorene‐alt‐(1,4‐phenylene‐(4‐sec‐butylphenyl)imino)‐1,4‐phenylene) (TFB) can offer a superior mobility of 1 × 10^−2^ cm^2^V^−1^s^−1^ to relieve the unbalanced carrier transport [20, 21], but they exhibit poor surface affinity toward perovskite precursors, leading to catastrophic film discontinuity. Therefore, it is highly expected to concurrently improve the interfacial quality and spin polarized carrier transport by HTL engineering toward high‐brightness spin‐LEDs for practical applications.

Following the first report of hybrid PVK:TFB‐based organic light‐emitting diodes (OLEDs) [22], this hybrid HTL has been sometimes adopted in pushing the efficiency of perovskite devices [23], and blue QLEDs [24, 25]. However, the fundamental transport mechanism within the PVK:TFB composite remains an unresolved “black box”. The exact nature of how charge carriers transport within the phase‐separated landscape and how energy‐level offsets are mitigated at the nanoscale has lacked direct experimental validation. Therefore, elucidating transport dynamics is urgently needed to harness multiple merits by the hybrid HTLs as to improve interfacial quality, form multiple high‐mobility pathways, smooth energy landscapes etc. which can hardly be achieved by single HTL.

In this work, a hybrid HTL engineering strategy is presented to resolve the fundamental “mobility‐compatibility” trade‐off for high brightness spin‐QLEDs. Tri‐channel hole transport mechanism is first delineated theoretically and experimentally to realize high‐mobility pathways, stepwise injection, and bypass the resistive limitations of traditional HTL. Moreover, within this phase‐separated framework of hybrid HTL, high‐energy nucleation sites facilitate the growth of a high‐quality chiral perovskite spin‐filter and lower the effective injection barrier. This architecture yields an ultralong T_50_ lifetimes to be 3968.9 h which is orders of magnitude higher than other previous reports. Moreover, a record brightness of 14,370 cd/m^2^ for red‐emitting spin‐QLEDs (see Table S2) achieving competitive EQE (6.68%) and *g*_CP‐EL_ (0.026) simultaneously. This finding provides an effective long‐lived and ultra‐high brightness enhancement strategy for the next generation spin‐optoelectronic devices.

## 2 Results and Discussion

**FIGURE 1:**
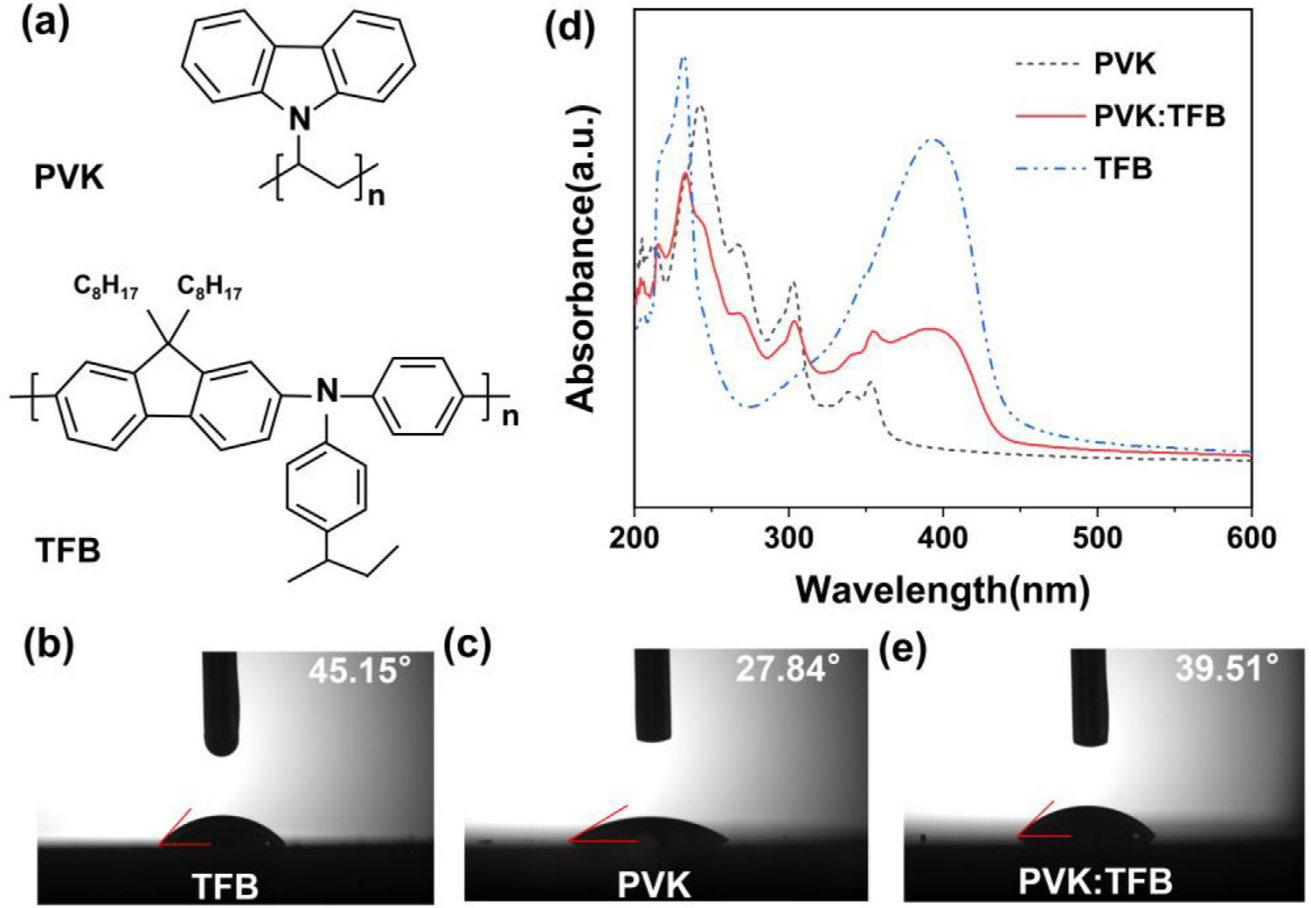
(a) Molecular structures of PVK and TFB. Contact angles of perovskite precursor solutions on (b) TFB and (c) PVK films. (d) Absorption spectra of the PVK, TFB, and PVK:TFB films. (e) Contact angle of perovskite precursor solution on a PVK:TFB film with a PVK:TFB weight ratio of 2:1.

The performance of spin‐QLEDs is inextricably linked to the structural and electronic properties of the HTL. Two HTL materials as PVK and TFB (molecular structures, Figure 1a) are used here and each has its own pros and cons resulting in “mobility‐compatibility” dilemma. From mobility point of view, TFB features a highly conjugated fluorene–triphenylamine backbone, facilitating efficient π‐π stacking and high hole mobility (1 × 10^−2^ cm^2^ V^−1^ s^−1^) [26]. Conversely, PVK utilizes a non‐conjugated ethylene main chain, which restricts charge transport to a significantly lower mobility of 2.5 × 10^−6^ cm^2^ V^−1^ s^−1^ [27, 28]. From interfacial quality point of view, TFB presents a significant challenge for the integration of polar perovskite layers. Chiral perovskite precursors dissolved in *N,N‐*Dimethylformamide (DMF) exhibit poor wettability on TFB surfaces (Figure 1b). This is fundamentally attributed to the surface migration of hydrophobic dioctyl side chains during film formation [29], which drastically reduces the surface free energy to a mere 15.7 mJ/m^2^ [30]. This low‐energy surface induces a high contact angle (45.15°) for the polar DMF‐based precursor, leading to discontinuous, “island‐like” nucleation (Figure S1b). Such morphological defects impede uniform spin‐polarized injection. In contrast, the absence of long alkyl chains in PVK results in a higher surface free energy (28.9 mJ/m^2^) [30]. yielding a significantly reduced contact angle of (27.84°) (Figure 1c). This enhanced hydrophilicity promotes the formation of continuous, pinhole‐free 2D perovskite films as shown in Figure S1a.

To harness the merits of both PVK and TFB and resolve this “mobility‐compatibility” contradiction for spin‐QLEDs, a hybrid PVK:TFB HTL strategy has been first proposed. UV–vis absorption spectra of the physical blend (Figure 1d) confirm that the characteristic electronic signatures of both components are preserved, indicating a lack of deleterious chemical interactions. By precisely tuning the PVK:TFB ratio, the interfacial energy and charge transport dynamics have been effectively modulated. The contact angle of the 2:1 PVK:TFB blend (39.51°) represents an optimized equilibrium between the extreme hydrophobicity of TFB and the favorable wetting of PVK (Figure 1e). Crucially, this hybrid surface facilitates the growth of a compact, high‐quality chiral perovskite film (Figure S1c). The 2:1 ratio provides a sufficient density of high‐energy PVK domains to ensure uniform precursor spreading, while the intercalated TFB domains provide high‐mobility pathways that bypass the resistive limitations of pure PVK. This dual‐functional architecture establishes a robust foundation for achieving high‐luminance, spin‐polarized electroluminescence.

**FIGURE 2:**
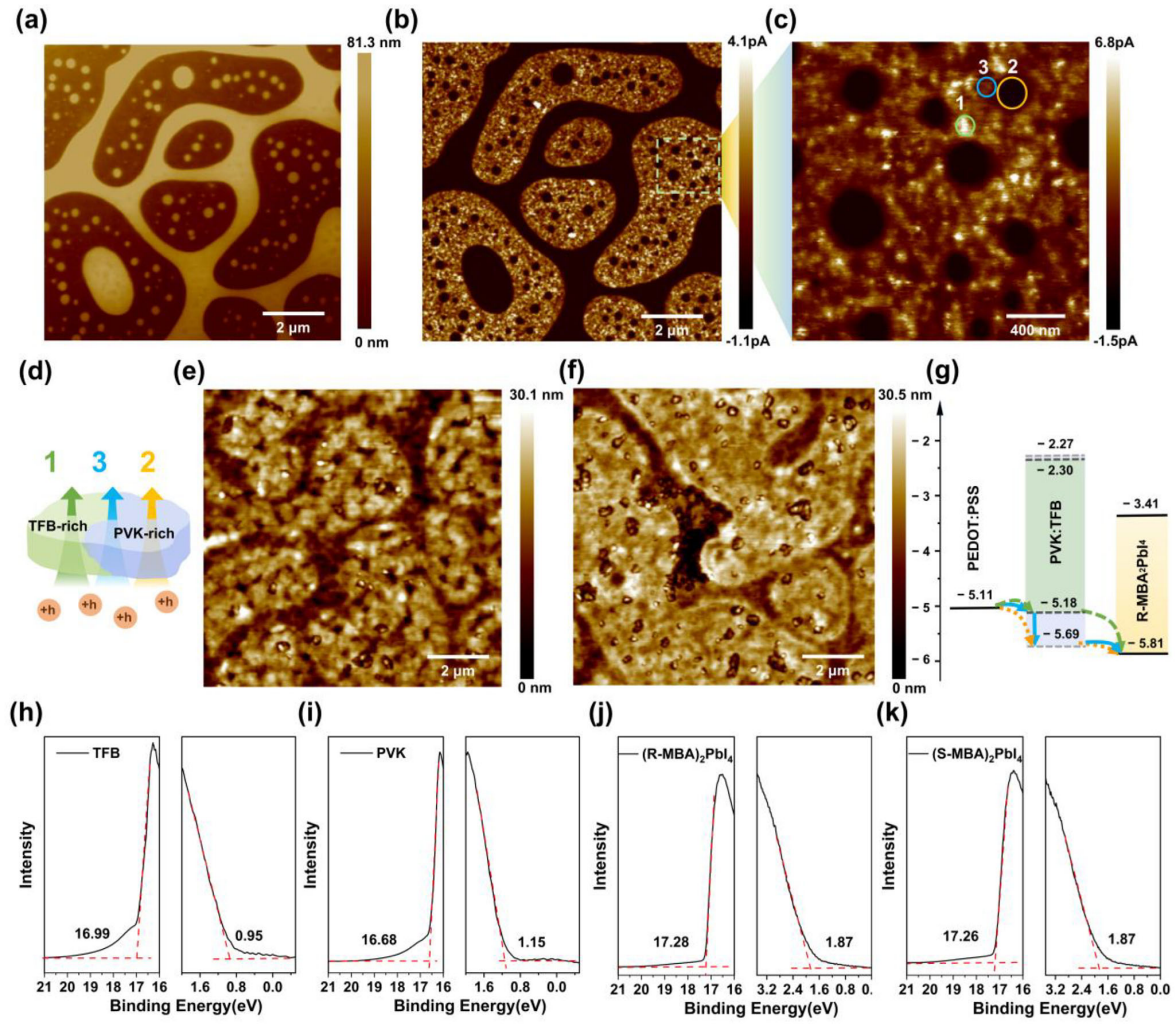
(a) AFM topography and (b) electrical current maps of PVK:TFB films on the glass/ITO/PEDOT:PSS substrates. In the AFM topography, the bright and dark regions correspond to PVK‐dominated and TFB‐dominated domains, respectively. (c) High (400 nm) magnifications of PVK:TFB films on the glass/ITO/PEDOT:PSS substrates. C‐AFM measurements were performed in contact mode at 1.0 V sample bias. (d) Schematics illustration of the three hole injection routes in the hybrid HTL layer. AFM topography of (e) (R‐MBA)_2_PbI_4_ and (f) (S‐MBA)_2_PbI_4_ films on the glass/ITO/PEDOT:PSS/PVK:TFB substrates. (g) Energy Levels of PEDOT:PSS/PVK:TFB/R‐MBA)_2_PbI_4_. UPS spectra of (h) TFB, (i) PVK, (j) (R‐MBA)_2_PbI_4_, and (k) (S‐MBA)_2_PbI_4_.

To elucidate the spatial distribution of the two polymers within the hybrid HTL, we employed conductive atomic force microscopy (C‐AFM) in contact mode. The topography of the 2:1 PVK:TFB film (Figure 2a) reveals a distinct microphase‐separated morphology, where the brighter and darker regions are correlated with PVK‐dominated and TFB‐dominated domains, respectively. Corresponding tunneling AFM (TUNA) current mappings (Figure 2b) confirm that this separation translates into a heterogeneous electronic landscape: the high‐conductivity “bright” areas correspond to TFB‐dominated phases (Area 1 in Figure 2c), while the lower‐conductivity “dark” regions represent PVK‐dominated domains (Area 2 in Figure 2c) (Figure S2a,b). However, as shown in the enlarged C‐AFM image in Figure 2c, there are brown areas (Area 3 in Figure 2c) with the conductivity lower than the bright “TFB” spots and higher than dark “PVK” islands (Figure S2c,d). As there are no new materials involved, the only reason could be such areas with the middle conductivity compared to pure “TFB” area and “PVK” area could be formed by the hybrid “PVK:TFB”, which means that TFB and PVK are not totally separated during the film deposition and could be hybrid as shown in Figure 2b,c. Therefore, there is a new hybrid “PVK:TFB” hole‐transport pathway besides the pure “TFB” and “PVK” channels which has been seldom reported (Figure 2d).

The (R/S‐MBA)_2_PbI_4_ crystals were synthesized via a refined crystallization protocol [18], resulting in high‐purity 2D chiral perovskite thin films. The chiral perovskite layers ((R/S‐MBA)_2_PbI_4_) exhibit excellent structural affinity for this phase‐separated template. The AFM images of chiral perovskite layers on the hybrid HTLs substrate are shown in Figure 2e,f. Upon deposition, we observed a dramatic “planarization” effect: the root–mean–square roughness (R_q_) decreased significantly from 19.5 nm (the bare hybrid HTL) to 3.79 and 3.31 nm (after deposition of (R‐MBA)_2_PbI_4_ and (S‐MBA)_2_PbI_4_ films), respectively (Figure S3a,c). This significant reduction in R_q_ indicates the formation of a continuous, high‐quality crystalline interface. The existence of the chiral perovskite further increases the resistance, which leads to the TUNA current maps with a low current signal shown in Figure S3b,d. No continued areas with higher or lower conductivity in the perovskite layer indicate that the chiral perovskite further confirms the continuous, well‐covered morphology of the perovskite layer on the PVK:TFB layer. Notably, no obvious macroscopic washing‐off or severe morphology disruption of the underlying PVK:TFB layer was observed after deposition of the DMF‐based perovskite precursor. The retained large‐scale morphological contours in Figure 2e,f suggest that the phase‐separated framework remains largely preserved.

The ultraviolet photoelectron spectroscopy (UPS) measurement has been used to quantify the energy level at these critical interfaces (Figure 2h–k). The highest occupied molecular orbital (HOMO) levels were calculated according to the following relation [31]: *HOMO* = *h*ν − [(*E*_cutoff_ − *E*_onset_)] where *hν* represents the incident photon energy (21.22 eV). The corresponding lowest unoccupied molecular orbital (LUMO) levels were derived from T_auc_ plots of the UV–vis absorption spectra (Table S1 and Figure S4). Our analysis reveals a synergistic energy cascade within the hybrid HTL architecture. The HOMO level of TFB (−5.18 eV) provides a smoother alignment with the PEDOT:PSS anode (−5.11 eV), facilitating low‐barrier hole extraction from the electrode. Simultaneously, the HOMO level of PVK (−5.69 eV) sits much closer to those of the (R‐MBA)_2_PbI_4_ and (S‐MBA)_2_PbI_4_ layers (−5.81 and −5.83 eV, respectively).

Based on the analysis of the spatial distribution and energy level of TFB and PVK within the hybrid HTL, two channels of hole transport are proposed here [25]. One channel is direct injection. Holes can transition directly from either TFB or PVK domains into the perovskite through tunneling or thermal activation. The other channel is stepwise injection in hybrid PVK:TFB area, in which the PVK serves as an energetic “stepping stone” that bridges TFB and the chiral perovskite, thereby smoothing the interfacial energy discontinuity and lowering the effective injection barrier (Figure 2g). Together, these parallel channels facilitate more efficient hole transport and contribute to the markedly enhanced electroluminescence performance.

**FIGURE 3:**
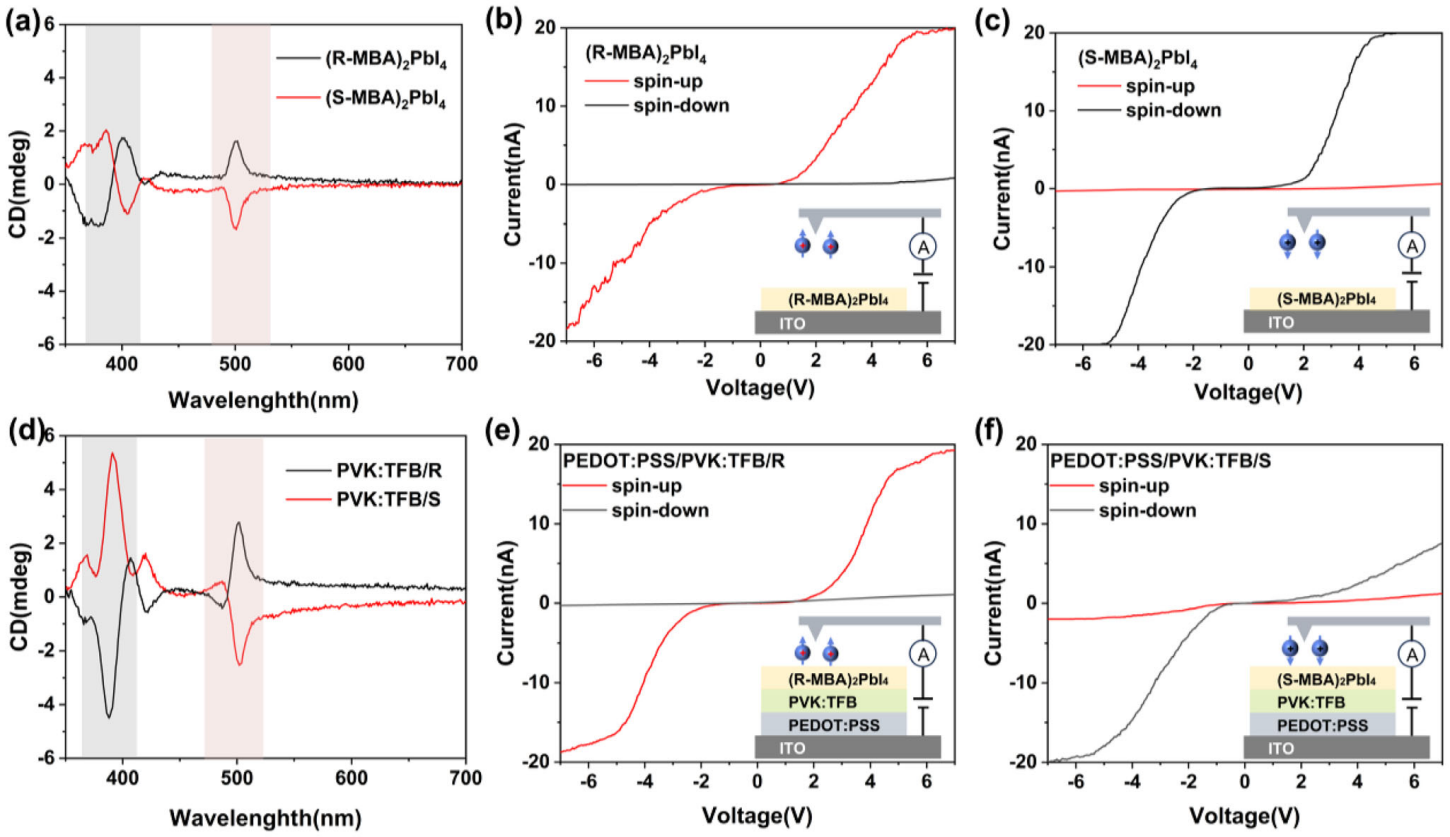
CD spectra of (a) (R/S‐MBA)_2_PbI_4_ and (d) (R/S‐MBA)_2_PbI_4_ on hybrid PVK:TFB. Room‐temperature *I–V* curves obtained using the mCP‐AFM technique of the chiral perovskite thin films for sample of (b) ITO/(R‐MBA)_2_PbI_4_, (c) ITO/(S‐MBA)_2_PbI_4_, (e) ITO/PEDOT:PSS/PVK:TFB/(R‐MBA)_2_PbI_4_, and (f) ITO/PEDOT:PSS/PVK:TFB/(S‐MBA)_2_PbI_4_, respectively.

To confirm the successful induction of molecular chirality into the inorganic frameworks, Circular dichroism (CD) characterization has been conducted. CD spectroscopy measures the differential absorption (Δ*A*) between left‐handed (LCP) and right‐handed (RCP) circularly polarized light, quantified as ellipticity (*θ*) through the relation [32]: $\theta \left(mdeg\right)=32980\times \left(A_{L}-A_{R}\right)$. The resulting spectra in Figure 3a,d reveals intense CD peaks at 375 nm and 493 nm, consistent with previous reports [19, 33]. Notably, both enantiomers exhibit a distinct sign inversion near 395 nm—a signature Cotton effect—which originates from the chiral‐induced splitting of electronic energy levels [34, 35].

To probe the fundamental spin physics of these films, we employed magnetic conductive probe atomic force microscopy (mCP‐AFM) using a Co/Cr‐coated tip [8, 10, 11, 36, 37, 38]. This technique directly measures the CISS effect by recording the current response under varied tip magnetization (N‐pole for *I*_spin‐up_ and S‐pole for *I*_spin‐down_). In these chiral systems, the “effective magnetic field” generated by the molecular inversion asymmetry filters charge carriers based on their spin‐associated magnetic moments [5]. The resulting spin polarization (*P*), defined as [10]: *P* = (*I*_spin‐up_ − *I*_spin‐down_)/(*I*_spin‐up_ + *I*_spin‐down_) × 100%, reaches a 95.4% for (R‐MBA)_2_PbI_4_ and −95.7% for (S‐MBA)_2_PbI_4_ at a bias of 6 V in Figure 3b,c, indicating that the synthesized chiral perovskite possesses the CISS effect.

A critical requirement for high‐performance spin‐QLEDs is that the chiral spin‐filter maintains its functionality when integrated onto the optimized HTL. We therefore evaluated the chiroptical properties of the perovskite films crystallized specifically on the PVK:TFB (2:1) substrate. The absorption and CD spectra in Figure S5b and Figure 3d remain highly consistent with the quartz‐based controls (Figure S5a). To account for film thickness variations and ensure a rigorous comparison, we calculated the absorption dissymmetry factor (g_abs_) [39]: *g*_abs_ = θ(mdeg)/(32980 × absorbance). The *g*_abs_ values for samples on the hybrid HTL are comparable to those on pure chiral perovskite films in Figure S5c, confirming that our HTL engineering strategy does not compromise chiral lattice formation. Furthermore, mCP‐AFM measurements on the HTL‐supported films (Figure 3e,f) yield a robust spin polarization of ±90%. This demonstrates that the hybrid PVK:TFB architecture not only facilitates superior film morphology but also preserves the high‐efficiency spin‐polarized transport necessary for state‐of‐the‐art spin‐optoelectronic devices.

**FIGURE 4:**
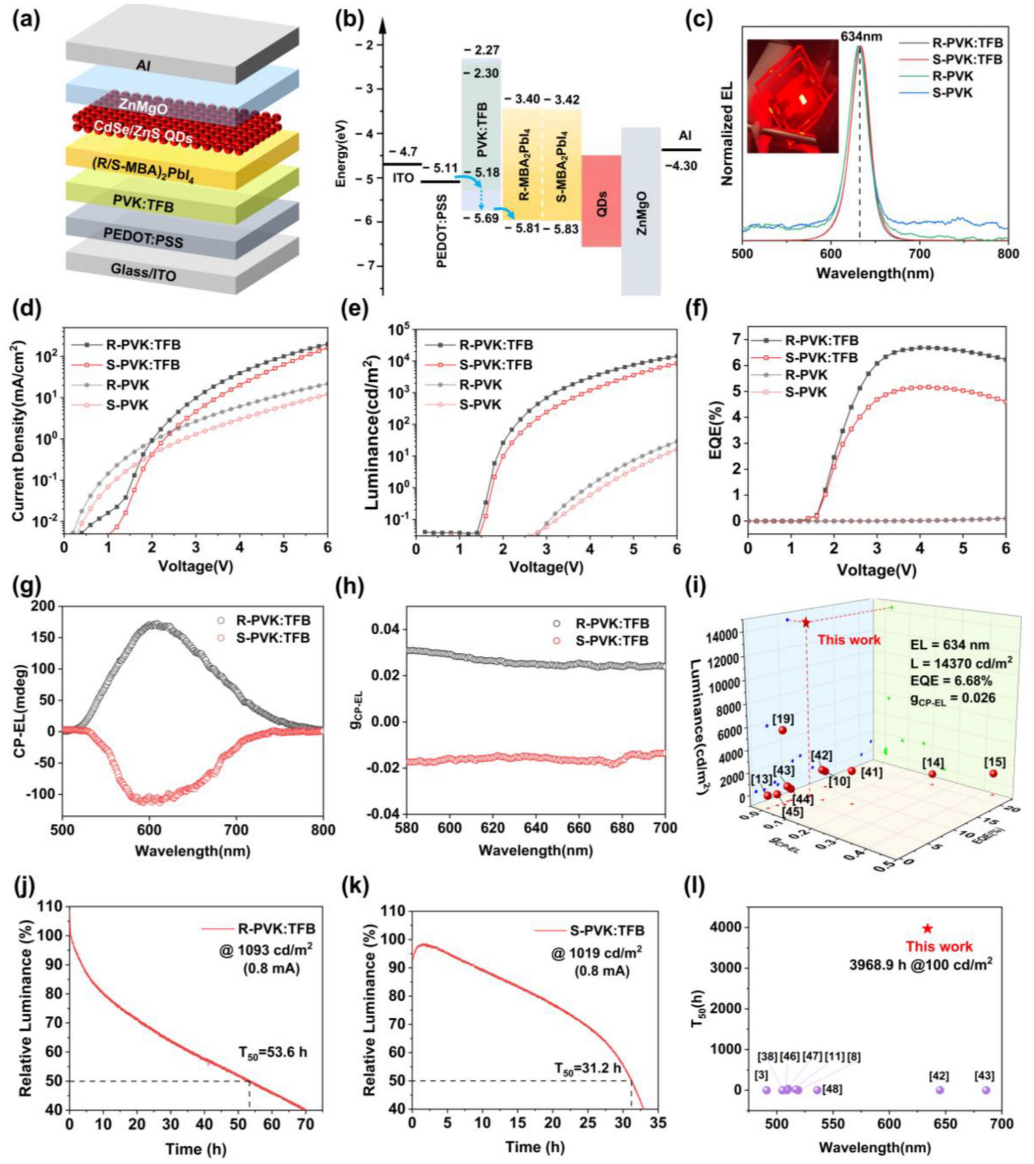
(a) Device structure, (b) energy‐level diagram of spin‐QLEDs based on the PVK:TFB HTL. (c) EL spectra, (d) current density–voltage characteristics, (e) luminance–voltage characteristics, and (f) EQE as a function of voltage. (g) CP‐EL degrees as a function of wavelength and (h) the corresponding *g*_CP‐EL_ values of spin‐QLEDs based on the PVK:TFB HTL. (i) Comparison of the performance of reported red spin‐LEDs [10, 13, 14, 15, 19, 41, 42, 43, 44, 45]. Time‐dependent electroluminescence intensity of (j) spin‐QLEDs‐R and (k) spin‐QLEDs‐S based on PVK:TFB under constant‐current operation at 0.8 mA, with initial luminances of 1093 and 1019 cd/m^2^, respectively. (l) Comparison of reported *T*_50_ lifetimes for spin‐QLEDs [3, 8, 11, 38, 42, 43, 46, 47, 48].

To evaluate the impact of interfacial engineering on device physics, we fabricated spin‐QLEDs with the architecture: ITO/PEDOT:PSS/HTL (PVK or PVK:TFB)/chiral perovskite/QDs/ZnMgO/Al (Figure 4a,b). Pure TFB was not employed as the HTL in the spin‐QLEDs because the chiral perovskite forms only sparse and discontinuous islands on TFB, leaving large areas of the underlying TFB exposed (Figure S1b). Such insufficient coverage prevents the formation of a continuous CISS‐active layer and allows carriers to bypass the chiral perovskite, thereby severely compromising the CP‐EL. All devices exhibited high‐purity red emission centered at 634 nm (Figure 4c), consistent with the EL spectrum of the reference QLED without the chiral perovskite layer, with the structure of ITO/PEDOT/TFB/QDs/ZnMgO/Al (Figure S6). The reference device exhibits higher maximum luminance and EQE, together with a slightly lower operating voltage, indicating that insertion of the chiral perovskite layer introduces moderate resistive and optical losses. Nevertheless, the chiral perovskite layer is indispensable for CISS‐based spin filtering and CP‐EL. The chiral 2D perovskite and the colloidal QDs are functionally decoupled. The chiral perovskite layer serves exclusively as a spin‐filter, utilizing the CISS effect to polarize the injected holes. The CdSe/ZnS QDs then act as the emissive layer, where these spin‐polarized holes recombine radiatively with electrons. The current density, luminance and external quantum efficiency (EQE) profiles reveal a great improvement in the hybrid HTL architecture (Figure 4d–f).

The transition from pure PVK to the PVK:TFB hybrid results in a dramatic reduction of the turn‐on voltage (*V*_on_, defined at a luminance of 1 cd/m^2^) from 4.0 to 1.7 V. This substantial decrease in the *V*_on_ coupled with a significantly higher current density at operating voltages, confirms the efficacy of the stepwise energy‐level alignment and the enhanced effective hole mobility provided by the TFB component. The EQE increased more than 30‐fold, reaching 6.68%. The peak luminance increased from 214 cd/m^2^ to a record‐breaking 14,370 cd/m^2^ for the red spin‐QLED‐R (Figure S7 and Table S2). Although the luminance continues to rise up to 6 V, the rate of increase (slope) begins to diminish between 5.8 and 6 V, suggesting that the device is approaching its optimal operating window. Pushing the voltage further would likely result in catastrophic dielectric breakdown rather than a meaningful increase in usable brightness. This performance represents the highest luminance reported to date for red‐emitting spin‐LEDs (Figure 4i). Similar enhancements were observed for the S‐enantiomer devices (8433 cd/m^2^; EQE 5.17%).

The scientific significance of these results lies in the resolution of the carrier‐injection imbalance. In standard QLEDs, the electron mobility of ZnMgO typically dwarfs hole mobility. In spin‐QLEDs, this disparity is traditionally “aggravated” by the resistive nature of the 2D perovskite spin‐filter [19]. The proposed hybrid HTL strategy effectively enhances the carrier transport and balances the charge carriers in the active layer. This architectural engineering demonstrates that high‐brightness spin‐optoelectronics are achievable by bridging the gap between transport mobility and interfacial compatibility.

Direct evidence of spin‐polarized transport is provided by the CP‐EL spectra (Figure 4g), which exhibit intense chiroptical signals centered at 634 nm, corresponding to the QD emission and further highlighting the distinct roles of the chiral perovskite in spin filtering and the QDs in light emission. The spectrometer quantifies this chiroptical activity through the ellipticity of the emitted light, which is derived from the intensities of the left‐handed (*I*_L_) and right‐handed (*I*_R_) circularly polarized components. The electroluminescence dissymmetry factor (*g*_CP‐EL_) is calculated using the standard relation [40]: *g*_CP‐EL_ = 2(*I*_L_ − *I*_R_)/(*I*_L_ + *I*_R_). Our spin‐QLEDs based on (R‐MBA)_2_PbI_4_ and (S‐MBA)_2_PbI_4_ demonstrate robust CP‐EL with mirror‐image helicity (Figure 4h). The maximum *g*_CP‐EL_ values reached 0.026 for the R‐enantiomer and −0.016 for the S‐enantiomer, a two‐fold enhancement over our previous work [19].

The robust *g*_CP‐EL_ can be understood as a combined consequence of efficient CISS spin filtering and improved utilization of spin‐polarized holes. The hybrid PVK:TFB HTL provides a stepwise energy‐level alignment that lowers the effective hole‐injection barrier and improves carrier transport and balance, facilitating the delivery of spin‐polarized holes toward the QD recombination region. Meanwhile, the improved interfacial compatibility of the hybrid HTL enables the formation of a continuous and well‐covered chiral‐perovskite spin‐filter, thereby reducing carrier bypass through uncovered non‐chiral regions. Importantly, the chiral perovskite on the hybrid HTL retains a spin polarization of approximately ±90%, indicating that its intrinsic CISS functionality is well preserved. These combined effects enable spin‐polarized holes to participate effectively in radiative recombination with electrons in the QDs, leading to the simultaneous achievement of high luminance, high EQE, and robust *g*_CP‐EL_.

The operational stability of the spin‐QLEDs was systematically evaluated under constant‐current driving conditions. The spin‐QLED‐R based on PVK:TFB exhibited a *T*_50_ lifetime of 53.6 h at an initial luminance of 1093 cd/m^2^, while the spin‐QLED‐S showed a *T*_50_ lifetime of 31.2 h at an initial luminance of 1019 cd/m^2^ (Figure 4j,k). To assess their stability under a standard operating luminance, the accelerated lifetime model, *L*_0_^*n*^ *T*_0_ = constant, was adopted, where *L*_0_ is the initial luminance and *n* is the acceleration factor. By using an acceleration factor of 1.8 [49], the *T*_50_ lifetimes at 100 cd/m^2^ were extrapolated to be 3968.9 h for the spin‐QLED‐R and 2036.4 h for the spin‐QLED‐S. These results demonstrate the superior operational stability of the present spin‐QLEDs, placing them among the most stable spin‐QLEDs reported to date (Figure 4l). The corresponding driving voltages were simultaneously monitored during the constant‐current aging measurements (Figure S8). After an initial electrical stabilization period characterized by a rapid decrease in the driving voltage, both devices exhibited a progressive voltage increase under constant‐current operation. This long‐term increase indicates a gradual increase in the effective device resistance, suggesting progressively deteriorated carrier injection and transport during aging.

**FIGURE 5:**
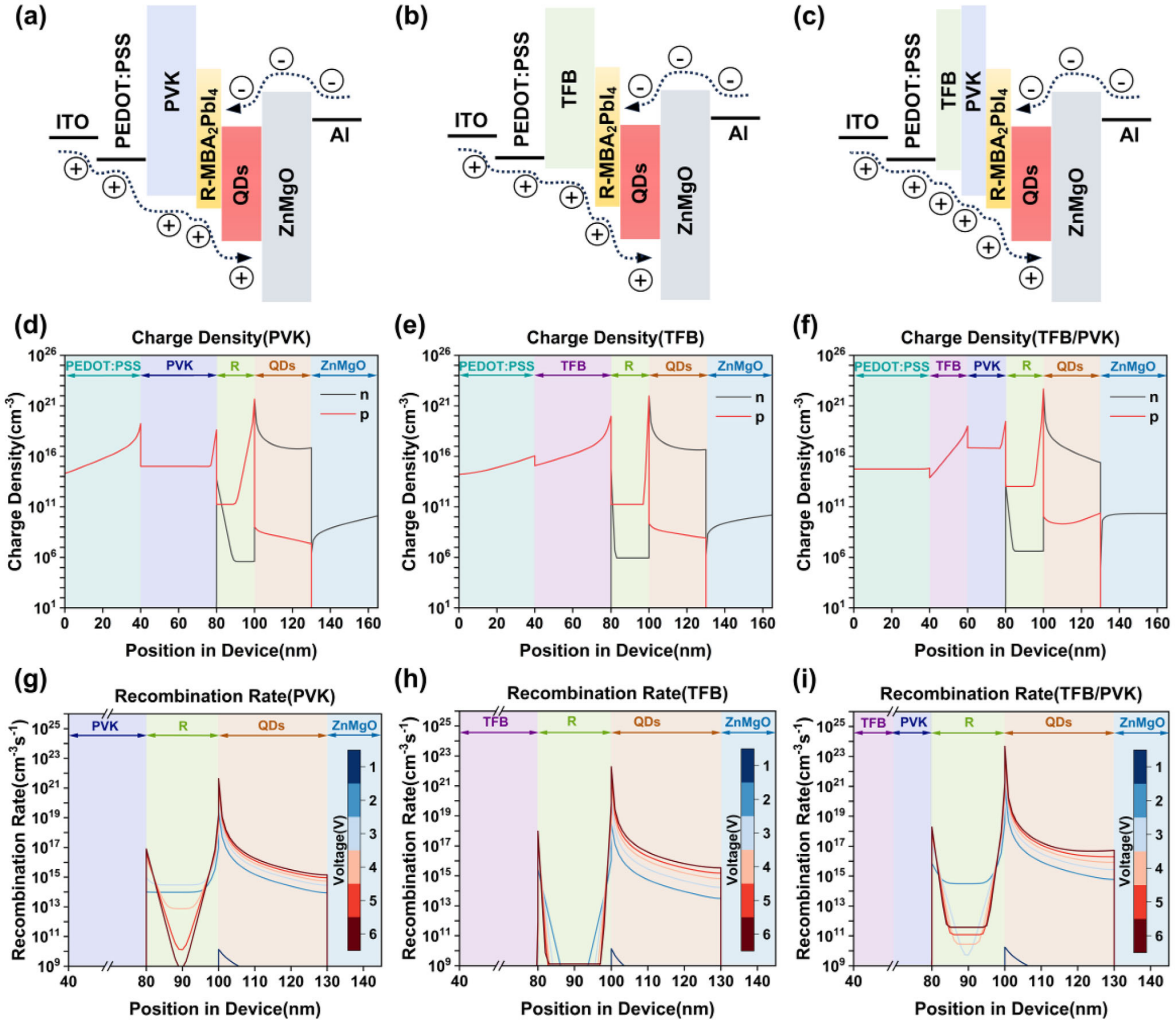
The energy band diagrams of devices with HTL configured as (a) PVK, (b) TFB, and (c) a bilayer of TFB and PVK; Diagrams of charge density distribution of spin‐QLEDs at the bias of 6 V with (d) PVK, (e) TFB and (f) TFB/PVK as the HTL; Diagrams of recombination rate of spin‐QLEDs with (g) PVK, (h) TFB and (i) TFB/PVK as the HTL.

To further support the above discussion on phase separation in the hybrid HTL leading to multiple carrier transport pathways, simulation models were constructed for three possible scenarios. The simulated device structure is ITO/PEDOT:PSS/HTL/(R‐MBA)_2_PbI_4_/QDs/ZnMgO/Al, where the HTL is composed of PVK, TFB, and a bilayer structure of TFB and PVK, respectively. Figure 5a–c presents the energy band diagrams of devices with different hole‐transport pathways. We employed a comprehensive drift‐diffusion model, coupling Poisson's equation with carrier continuity equations. To accurately reflect the energetic and spatial disorder intrinsic to semiconducting polymers [50, 51], we utilized the Extended Gaussian Disorder Model (EGDM) to describe carrier mobilities [52, 53]. All detailed parameters are listed in Table S3. Rather than being mutually exclusive, these models, including monolithic PVK, monolithic TFB, and a TFB/PVK “stepped” architecture, characterize the diverse local environments charge carriers encounter.

In regions dominated by PVK, the high LUMO level provides an exceptional electron‐blocking capability. While the large energetic barrier at the PEDOT:PSS interface and lower mobility result in a “bottleneck” effect (Figure 5a,d), this pathway is vital for confining electrons within the emissive zone. The recombination rate in this regime (Figure 5g) remains highly localized at the target interface, effectively suppressing non‐radiative losses deeper in the HTL.

TFB‐dominated domains function as high‐mobility highways. TFB facilitates efficient hole extraction from the anode and, despite the single‐step barrier at the perovskite interface (Figure 5b), achieves a significantly higher recombination rate of 1.92 ×10^22^ cm^−3^s^−1^ at the recombination zone compared to pure PVK (Figure 5h). This pathway is responsible for maintaining the high current density required for high‐brightness operation (Figure 5e). The simulated hybrid (bilayer) configuration effectively decomposes the prohibitive 0.63 eV barrier into two manageable steps (0.51 eV and 0.12 eV) (Figure 5c). This graded energetic landscape, facilitated by the high‐mobility TFB backbone and the buffer‐like PVK domains, homogenizes the hole distribution throughout the device (Figure 5f). The primary recombination rate at the 100 nm emissive zone is bolstered to 4.83 × 10^23^ cm^−3^ s^−1^ (Figure 5i). These results underscore a vital design principle for spin‐optoelectronics: device efficiency is dictated not by the total injection barrier, but by the maximum single‐step barrier height and the mobility compatibility between sublayers. A thickness scan of the TFB component further confirms that increasing the high‐mobility fraction while maintaining interfacial compatibility can push recombination rates as high as 1.26 × 10^24^ cm^−3^s^−1^ (Figure S9). By integrating these pathways, the hybrid HTL ensures that the maximum population of spin‐polarized holes is delivered to the recombination zone with minimal carrier loss and reduced “dwell time” at dissipative interfaces.

## 3 Conclusion

In summary, this work establishes a hybrid HTLs engineering strategy to achieve ultra high‐brightness and most long‐lived spin‐QLEDs. Through demonstrating a hybrid HTL of PVK:TFB, we have successfully achieved the two key merits of high interfacial quality and high‐mobility spin‐polarized charge transport. As confirmed by contact angle measurements and morphological analysis, the PVK component enhances substrate surface energy, ensuring the formation of a continuous chiral perovskite spin‐filter, which is a feat unattainable on pure, hydrophobic TFB surfaces. Meanwhile, the intercalated TFB network maintains high effective hole mobility, as evidenced by *J–V–L* characteristics and C‐AFM current mapping. Most importantly, a third spin‐polarized transport channel is first discovered as a hybrid TFB:PVK channel, where PVK acts as an energetic “stepping stone” that bridges the abrupt HOMO‐level discontinuity between TFB and the perovskite, effectively smoothing the injection barrier and accelerating carrier transport. The resulting spin‐QLEDs demonstrated an ultralong *T*_50_ lifetime at 100 cd/m^2^ to be 3968.9 h which is orders of magnitude higher than other reports. Moreover, the spin‐QLEDs exhibit a recorded luminance of 14,370 cd/m^2^ for red‐light‐emitting devices, alongside an external quantum efficiency of 6.68% and a *g*_CP‐EL_ of 0.026. Despite the high spin polarization of approximately 90% retained by the chiral perovskite on the hybrid HTL, the *g*_CP‐EL_ remains limited by spin losses occurring after CISS spin filtering. Interfacial depolarization during carrier transfer and spin relaxation in the QDs prior to radiative recombination are therefore considered important loss channels, highlighting interface optimization and spin preservation in the emissive region as key directions for further improvement. This work provides a reliable framework for optimizing injection in chiral electroluminescent systems and offers a pathway for long lifetime and high‐brightness applications.

## 4 Methods

### 4.1 Materials

R‐𝛼‐methylbenzylamine (R‐MBA, TCI), S‐𝛼‐methylbenzylamine (S‐MBA, TCI), lead iodide (PbI_2_, TCI), hydriodic acid (HI, 55–58 wt% in aqueous solution, Aladdin Ltd, Shanghai, China), hypophosphorous acid (H_3_PO_2_, MACKLIN Company, Shanghai, China), N,N‐dimethylformamide (DMF, 99%, Sigma–Aldrich), chlorobenzene(CB, anhydrous, 99.5%, Aladdin Ltd, Shanghai, China), CdSe/ZnS QDs and zinc magnesium oxide (ZnMgO) nanocrystals were purchased from Suzhou Xingshuo Nanotech Co., PEDOT:PSS (Luminescence Technology Corp., Taiwan, China), TFB (Montreal Optoelectronics Inc.), and PVK (P‐OLED Technology Company, Shanghai, China).

### 4.2 Synthesis of (R/S‐MBA)_2_PbI_4_ Crystals

2.305 g (5 mmol) of PbI_2_ was dissolved in a mixture of 10mL HI and 5 mL H_3_PO_2_ in a flask and heated with stirring at a temperature of 90°C. Separately, 1.289 mL (10 mmol) of R/S‐MBA was slowly added dropwise into 5 mL HI in another beaker and stirred in an ice bath for 3 h to form the R/S‐MBAI solution. This solution was then added to the flask, and the reaction mixture was stirred at a temperature of 130°C for 2 h. After reaction completion, the solution was cooled naturally to room temperature for crystallization. The echinoid‐like orange crystals were collected by filtration, washed with diethyl ether, and dried under vacuum at 60°C overnight.

### 4.3 Device Fabrication

The devices were fabricated on the patterned ITO‐glass substrates with a sheet resistance of 20 Ω per square. Patterned ITO glass was sonicated in detergents, deionized water, ethanol, and deionized water for 15 min consecutively. The cleaned substrate was blown by the nitrogen and dried at 120°C for 10 min, and then treated with plasma cleaner for 5 min. PEDOT:PSS (filtrated before use) was spin‐coated on the substrate at 3000 rpm for 45 s, followed by 130°C drying on a hotplate for 15 min. Then the substrate was transferred into a glovebox filled with nitrogen. The hybrid HTL is prepared by thoroughly blending PVK and TFB at a weight ratio of 2:1, followed by dissolving the mixture in chlorobenzene with a concentration of 10 mg mL^−1^. The HTL solution (PVK or PVK:TFB blend) was spin‐coated at 3000 rpm and then annealed at 100°C for 10 min. The chiral perovskite precursor solution (0.05 mol L^−1^ in DMF) was subsequently spin‐coated at 3000 rpm, followed by annealing at 100°C for 10 min to form the perovskite layer. Next, the CdSe/ZnS QDs solution (17 mg m^L‐1^ in n‐octane) was spin‐coated at 3000 rpm and annealed at 100°C for 5 min. The ZnMgO layer (25 mg mL^−1^ in ethanol) was then deposited by spin coating at 3000 rpm and annealed at 100°C for 10 min. 100 nm Al was then evaporated under a pressure of 5 × 10^−4^ Pa. The as‐prepared device was encapsulated before further characterization.

### 4.4 Characterization

The UV–vis absorption spectra were recorded by PERSEE TU‐1901 spectrometer. The CD were measured by Chirascan V100. AFM images were collected by Bruker Dimension icon. The UPS measurements were conducted on the Thermo ESCALAB XI+. The current density–voltage–luminance–EQE (J–V–L–EQE) of the device were measured by a LED characterization system XPQY‐EQE‐350‐1100 (Guangzhou Xi Pu Optoelectronics Technology Co., Ltd.) combined with an integrated sphere GPS‐4P‐SL (Labsphere), a spectrometer USB2000+ (Ocean Optics), and a photodetector S7031‐1006 (Hamamastu Photonics). The CP‐EL was measured by JASCO CPL‐300 spectrophotometer with external bias.

### 4.5 Numerical Simulation

Electrical simulations were performed using Setfos 5.5 software. The parameters of each layer material used in the simulations, including thickness, highest occupied molecular orbital (HOMO) energy level, lowest unoccupied molecular orbital (LUMO) energy level, work function (*W*_F_), electron mobility (*µ*_e_) and hole mobility (*µ*_h_), are listed in the Table S3. The carrier mobilities of TFB and PVK are described using the extended Gaussian disorder model (EGDM), while the carrier mobilities of other material were set to constant values. Defect states were ignored, and only Langevin recombination was taken into account. Since the injection barriers at both the ITO/PEDOT:PSS interface and Al/ZnMgO interface are relatively high, the carrier injection type at these two interfaces was set to thermionic emission. Finally, the charge drift‐diffusion model was used to simulate the electrical properties of the spin quantum dot light‐emitting diode.

## Author Contributions

**Fengqi Qiu**: investigation, Writing – original draft, formal analysis. **Hechun Zhang**: investigation, writing – original draft, formal analysis. **Wenbo Liu**: writing – review and editing, resources, investigation, supervision, methodology. **Guiming Zhong**: investigation, formal analysis. **Fumin Lu**: investigation, formal analysis. **Yuqi Wang**: investigation, formal analysis. **Kai Wang**: methodology, writing – review and editing, project administration, resources, supervision. **Dan Wu**: project administration, supervision, resources, writing – review and editing, methodology. **Fujin Ai**: resources, data curation.

## Funding

This work was supported by the National Natural Science Foundation of China (Nos. 62475171, 62122034, and 62404091), Guangdong Basic and Applied Basic Research Foundation (No. 2025A1515011655), Young Top Talents under Guangdong Special Support Program (No. 2024TQ08×142), Guangdong Major Project of Basic Research (No. 2025B0303000002), Shenzhen Science and Technology Program (20231128135041001), and Shenzhen Key Laboratory for Advanced Quantum Dot Displays and Lighting (No. SYSPG20241211173725009), and Guangdong Provincial Key Laboratory of Low‐Altitude Intelligent Perception and Navigation Safety (No. 2026B1201010001).

## Conflicts of Interest

The authors declare no conflicts of interest.

## Supporting information




**Supporting File**: advs77899‐sup‐0001‐SuppMat.docx.

## Data Availability

The data that support the findings of this study are available from the corresponding author upon reasonable request.
