# Supplementary material for: High‐Brightness and Ultra‐Long‐Lifetime Spin Quantum Dot Light‐Emitting Diodes Enabled by Efficient Injection of Spin‐Polarized Holes Through a Hybrid Hole Transport Layer
Source: Adv Sci (Weinh). 2026 Sep 27:e77899. Online ahead of print. doi: 10.1002/advs.77899 (PMC13616287; doi:10.1002/advs.77899)
Supplement: Supplementary file 1 [file ADVS-9999-e77899-s001.docx]

Supporting Information

High-Brightness and Ultra-Long-Lifetime Spin Quantum Dot Light-Emitting Diodes Enabled by Efficient Injection of Spin-Polarized Holes through a Hybrid Hole Transport Layer

Fengqi Qiu, Hechun Zhang, Wenbo Liu*, Guiming Zhong, Fumin Lu, Yuqi Wang, Fujin Ai, Kai Wang*, and Dan Wu*

**Figure S1.** Photograph of chiral perovskite coverage on (a) PVK films, (b) TFB films and (c) PVK:TFB films with the ratio of 2:1. All the substrate sizes are 2.5 cm × 2.5 cm. In (b), the chiral perovskite forms only sparsely distributed islands, while the green regions correspond to the exposed TFB surface.

**Figure S2.** (a) C-AFM topography and (b) electrical current maps of PVK on ITO/PEDOT:PSS. (c) Randomly chosen four cross-sectional lines in C-AFM topography of PVK:TFB on ITO/PEDOT:PSS in Figure 2c. (d) The corresponding cross-sectional current distribution of the four lines. Area 1 is for the TFB conduction channel. Area 2 is for the PVK conduction channel. Area 3 is for the PVK:TFB conduction channel.

**Figure S3.** 3D AFM topography of (a) (R-MBA)_2_PbI_4_ and (c) (S-MBA)_2_PbI_4_ films on the glass/ITO/PEDOT:PSS/ PVK:TFB substrates. Electrical current maps of (b) (R-MBA)_2_PbI_4_ and (d) (S-MBA)_2_PbI_4_ films on the glass/ITO/PEDOT:PSS/ PVK:TFB substrates.

**Figure S4.** UPS spectra of (a) PEDOT:PSS and (b) CdSe/ZnS QDs. T_auc_ plots of (c) TFB, (d) PVK, (e) (R-MBA)_2_PbI_4_, and (f) (S-MBA)_2_PbI_4_.

**Figure S5.** Absorption spectra of (a) (R/S-MBA)_2_PbI_4_ and (b) (R/S-MBA)_2_PbI_4_ on hybrid PVK:TFB and (c) the calculated absorption dissymmetry factors as a function of wavelength.

**Figure S6.** (a) EL spectra, (b) EQE–voltage and (c) current density–voltage–luminance characteristics of QLED with the structure of ITO/PEDOT:PSS/TFB/QDs/ZnMgO/Al.

**Figure S7.** Comparisons of red spin-LEDs’ luminance.

**Figure S8.** Evolution of the driving voltage of (a) spin-QLED-R and (b) spin-QLED-S during constant-current aging measurements at 0.8 mA. The voltage was recorded simultaneously with the operational lifetime measurements.

**Figure S9.** Simulation of carrier recombination rate distribution of the spin-QLEDs at the bias of 6V.

**Table S1.** Energy level parameters of functional layers determined from UPS and optical absorption (Tauc) analyses.

| Layer | E_cutoff_(eV) | E_onset_(eV) | E_VBM_(eV) | E_g_(eV) | E_CBM_(eV) |
| --- | --- | --- | --- | --- | --- |
| PEDOT:PSS | 16.69 | 0.58 | −5.11 |  |  |
| PVK | 16.68 | 1.15 | −5.69 | 3.42 | −2.27 |
| TFB | 16.99 | 0.95 | −5.18 | 2.88 | −2.30 |
| (R-MBA)_2_PbI_4_ | 17.28 | 1.87 | −5.81 | 2.41 | −3.40 |
| (S-MBA)_2_PbI_4_ | 17.26 | 1.87 | −5.83 | 2.41 | −3.42 |
| CdSe/ZnS QDs | 17.96 | 3.17 | −6.43 |  |  |

**Table S2.** Comparison of performance parameters of spin-LEDs.

| **Color** | **Device Structure** | **Spin Filter Layer** | **Emitting Layer** | **Material Type** | **Emission Peak**  **(nm)** | **\|g_CP-EL_\|** | **L**  **(cd/m^2^)** | **EQE**  **(%)** | **V_on_**  **(V)** | **T_50_** | **Year** | **Ref.** |
| --- | --- | --- | --- | --- | --- | --- | --- | --- | --- | --- | --- | --- |
| Red | **ITO/PEDOT:PSS/PVK:TFB/Chiral layer/EML/ZnMgO/Al** | **(R/S-MBA)_2_PbI_4_** | **CdSe/ZnS** | **2D/QDs** | **634** | **2.6×10^−2^** | **14370** | **6.68** | **1.7** | **3968.9 h @100 cd cm^-2^** | **This work** | |
|  | ITO/PEDOT:PSS/EML/POT2T/LiF/Al | R/S-MBA_1–x_POEA_x_)_2_(MA_0.4_Cs_0.6_)_n−1_Pb_n_I_3n+1_ | | Quasi-2D | 696 | 5.4×10^−3^ | 46.36 | 4.22 | 2.5 | / | 2025 | [1] |
|  | ITO/m-PEDOT:PSS/EML/TPBi/LiF/Al | S-MBA with S-BHP in (R-/S-MBA)_1.5_(PEA)Cs_2_Pb_3_(I_0.7_Br_0.3_)_10_ | | Quasi-2D | 645 | 7.5×10^−2^ | 1753 | 6.9 | ~3 | 72 min @135 cd cm^-2^ | 2025 | [2] |
|  | ITO/NiOx/SAM/PVK/R-CHP/MoO_3_/Ag | (R/S-p-F-MBA)_2_Cs_1_FA_1_Pb_3_I_10_ | | Quasi-2D | 725 | 1.63×10^−1^ | ~2000 | 8.3 | 2.4 | / | 2025 | [3] |
|  | FITO/SAM/Chiral-MOF/EML/ZnO/Ag | D/L-Eu(Tar) | CdSe/ZnS | Chiral-MOF/QDs | 632 | 4.37×10^-1^ | ~1000 | 22.45 | 1.2 | / | 2025 | [4] |
|  | ITO/PEDOT:PSS /PTAA /PVK/EML/TPBi/Liq/Al | R/S-NEA_2_(FA_0.8_MA_0.2_)_2_Pb_3_Br_1_I_9_ | | Quasi-2D | 764 | 1.48×10^-2^ | ~10 | 10.5 | ~3.5 | / | 2025 | [5] |
|  | ITO/PEDOT:PSS/TFB/Chiral layer/EML/ZnO/Ag | (R/S-MBA)_2_PbI_4_ | CdSe/ZnS | 2D/QDs | 634 | 1.6×10^-2^ | 5638 | 2.7 | 1.9 | / | 2024 | [6] |
|  | ITO/SAM/Chiral-MOF/EML/ZnO/Ag | [Sr(9,10-adc)(DMAc)_2_]_n_ | CdSe/ZnS | Chiral-MOF/QDs | 642 | 2.45×10^-1^ | 10* | 20.39 | 1.73 | / | 2023 | [7] |
|  | ITO/PEDOT:PSS/EML/TPBi/LiF/Al | (PEA_x_NEA_1-x_)_2_MA_n-1_Pb_n_I_3n+1_ | | Quasi-2D | 686 | 4.0×10^-3^ | 370 | 3.7 | 3.4 | 109 s @10 mA cm^-2^ | 2023 | [8] |
|  | ITO/PEDOT:PSS/TFB/EML/ZnO/Al | CdSe/CdS | | quantum rods | 600 | 2×10^-4^ | 135 | 0.5 | 2.6 | / | 2022 | [9] |
|  | ITO/m-PEDOT:PSS/Chiral layer/EML/TPBi/LiF/Al | (R/S-MBA)_2_PbI_4_ | CsPbI_3_ | 2D/NCs | 688 | 5.0×10^-3^ | 10.53 | 477 | 2.4 | / | 2021 | [10] |
| Green | ITO/m-PEDOT:PSS/EML/ ZADN/LiF/Al | CsPbBr_3_–BINAPO | | Core–Shell QDs | 510 | 12.4×10^-1^ | 10692 | 12.35 | 2 | 30.56 h @100 cd cm^-2^ | 2026 | [11] |
|  | ITO/PEDOT:PSS poly-TPD/ PF8Cz/EML/PO-T2T/LiF/ Al | R/S- CsPbBr_3_ NCs | | Chiral-NCs | 519 | 2.16×10^-3^ | 12800 | 15.4 | 2.5 | 20 min @1000 cd cm^-2^ | 2025 | [12] |
|  | ITO/PEDOT: PSS/EML/TPBi/LiF/Al | (R-/S-MBA)_2_FA_n-1_Pb_n_Br_3n+1_ | | Quasi-2D | 535 | 4.98×10^-2^ | ~500 | 10.4 | 3.7 | / | 2025 | [13] |
|  | ITO/poly-SAM/EML/PO-T2T/TPBi/LiF/Al | (R/S-MBA)chiral CsPbBr_3_ PQDs | | Chiral-NCs | 517 | 2.85×10^-1^ | 28000 | 16.8 | 2.1 | 19.8 h @100 cd cm^-2^ | 2025 | [14] |
|  | ITO/PEDOT:PSS/Chiral layer/EML/TPBi/LiF/Al | (R/S-MBA)_2_Pb(I_4-x_Br_x_) | Super Yellow | 2D/Organic polymer | ~550 | 1×10⁻² | ~10000 | 2 | ~5 | / | 2025 | [15] |
|  | ITO/PEDOT:PSS/EML/TPBi/LiF/Al | (t-BA)_2_(R/S-MBA)_2_MA_0.2_Cs_0.8_Pb_2_Br_9_ | | Quasi-2D | 510 | 1.03×10^-1^ | ~4800 | 3.6 | 4.3 | / | 2024 | [16] |
|  | ITO/NiOx/PTAA/PVK/EML/TPBi/LiF/Al | R/S-NEA_2_(FA_0.85_Cs_0.15_)_2_Pb_3_Br_10_ | | Quasi-2D | 530 | 7.8×10^-2^ | 1084 | 13.5 | 2.8 | / | 2024 | [17] |
|  | ITO/PEDOT:PSS/EML/TPBi/LiF/Al | (R/S-MBA)_2_(PEA)_2-X_MACsPb_3_Br_10_ | | Quasi-2D | 518 | 5.1×10^-2^ | 9300 | 3.8 | 4 | / | 2024 | [18] |
|  | ITO/PEDOT:PSS/ PVK/EML/TPBi/LiF/Al | CSA@MBA-Doped FAPbBr_3_ NCs | | Chiral-NCs | 509 | 2×10^–3^ | 510 | 3.1 | ~4 | 108 s @6.25 mA cm^-2^ | 2024 | [19] |
|  | ITO/PEDOT:PSS/EML/TpBi/LiF/Al | D/L-TBe_x_PEA_2-x_FA_2_Pb_3_Br_10-x_(BF_4_)_x_ | | CILs | 530 | 2.6×10^-1^ | 3808 | 12 | 3.4 | / | 2024 | [20] |
|  | ITO/PVK/EML/TPBi/LiF/Al | (R/S-MBA)_2_FA_2_Pb_3_Br_10_ | | Quasi-2D | 536 | 9.96×10^-2^ | 4800 | 15.42 | 2.8 | 2300 s @100 nits | 2024 | [21] |
|  | ITO/PEDOT:PSS/Chiral layer/EML/TPBi/LiF/Al | (S/R-BA)_2_PbBr_4_/I_4_ | Super Yellow | 2D/Organic polymer | 544-576 | 2.6×10^-2^ | 16803 | 3.6 | ~3.2 | / | 2024 | [22] |
|  | FTO/NiOx/doped poly-TPD/Chiral layer/EML/PO-T2T/LiF/Al | (PEA)_x_(S/R-PRDA)_2–x_Sn_0.1_Pb_0.9_Br_4_ | CsPbBr_3_ | 2D/NCs | 518 | 1.1×10^-3^ | 2500 | 5.7 | 2.2 | / | 2024 | [23] |
|  | Au/IZO/TFB/Al_2_O_3_/Chiral layer/EML/Au | (R/S-MBA)_2_PbI_4_ | (Al_0.32_Ga_0.68_)_0.5_In_0.5_P | 2D/MQWs | 590 | 3.0×10^-1^ | _ | <0.1 | _ | / | 2024 | [24] |
|  | ITO/PEDOT:PSS/EML/TPBi/LiF/Al | (R/S-NEA)/CsPbBr_3_ | | Core–Shell QDs | 505 | 2.4×10^-1^ | 1962 | 5.47 | 2.2 | 8.3 min @~30 mA cm^-2^ | 2023 | [25] |
|  | ITO/PEDOT:PSS/poly-TPD/EML/TPBi/LiF/Al | R/S-BinapO-passivated MAPbBr_3_ | | Chiral-NCs | 523 | 1.8×10^-1^ | 18783 | 3.6 | 3.2 | / | 2023 | [26] |
|  | ITO/PEDOT:PSS/TFB/EML/PMMA/TPBi/LiF/Al | (R/S-PEA)/MAPbBr_3_ | | Core–Shell QDs | ~520 | 6.0×10^-3^ | ~1100 | 3.6 | 3 | / | 2022 | [27] |
|  | ITO/PEDOT:PSS/PVK/EML/TPBi/LiF/Al | (2-PEA)_x_(R/S-2-BA)_2_MA_1_Cs_1_Pb_3_Br_10+x_ | | Quasi-2D | 515 | 5.5×10^-2^ | 21240 | 5.21 | 2.9 | / | 2024 | [28] |
| Blue |  |  |  |  | 465 | 1.22×10^-1^ | 517 | 1.51 | 3.4 |  |  |  |
|  |  |  |  |  | 442 | 1.75×10^-1^ | 132 | 0.94 | 3.5 |  |  |  |
|  | ITO/m-PEDOT:PSS/EML/TPBi/LiF/Al | L/D-TBe_x_(PEA_0.6_iPA_0.4_)_2-x_Cs_2_Pb_3_Br_10-x_(BF_4_)_x_ | | CILs | 491 | 1.58×10^-1^ | 1592 | 12.97 | 3 | > 500 s @10 mA cm^-2^ | 2025 | [29] |

**Table S3.** The parameters used in electrical and optical simulation.

| Layer | Thickness (nm) | HOMO (-eV) | LUMO (-eV) | W_F_ (eV) | μ_e_ (cm^2^/Vs) | μ_h_ (cm^2^/Vs) | ε @634nm |
| --- | --- | --- | --- | --- | --- | --- | --- |
| ITO | 100 |  |  | 4.70 |  |  |  |
| PEDOT:PSS^[30]^ | 40 | 5.11 | 2.81 |  |  | 2.60×10^−4^ | 2.25 |
| PVK^[31]^ | 40/20 | 5.69 | 2.27 |  |  | μ*=1.56×10^−3^ σ=0.124eV | 2.60 |
| TFB^[32]^ | 40/20 | 5.18 | 2.30 |  |  | μ*=1.90×10^−1^ σ=0.066eV | 2.82 |
| (R-MBA)_2_PbI_4_ | 20 | 5.81 | 3.42 |  | 2.30×10^−2^ | 2.30×10^−3^ | 5.56 |
| CdSe/ZnS QDs | 30 | 6.43 | 4.40 |  | 1.00×10^−3^ | 1.00×10^−3^ | 4.00 |
| ZnMgO^[33]^ | 40 | 7.60 | 3.80 |  | 2.00×10^1^ |  | 3.22 |
| Al | 100 |  |  | 4.30 |  |  |  |

Extended Gaussian disorder model (EGDM) is derived from the Pasveer mobility law and the generalized Einstein relation.^[34]^ The field- and temperature-dependent mobility based on EGDM is given by the following equation:^[35]^

where

Here, $\mu_{0}\text{（}T\text{）}$is the zero-field intrinsic mobility at low carrier concentration, further determined by the reference mobility ($\mu*$) and the degree of energetic disorder, expressed as $\mu_{0}(T)=\mu*\exp(-c_{2}\hat{\sigma}^{2})$, where $\hat{\sigma}=\sigma/(k_{\text{B}}T)$.The parameter *σ* is the width of the Gaussian density of states (DOS), representing the degree of energetic disorder, and *c*_2_=0.39 is a typical orbital overlap factor for polymeric materials. The parameter *a* denotes the characteristic hopping distance between localized transport sites, and N_0_ is the density of these sites. *g*_1_(*T*,*p*) accounts for the enhancement of mobility due to carrier concentration, while *g*_2_(*T*,*E*) describes the field-enhancement effect following a Poole–Frenkel-type mechanism, which reflects electric-field-induced lowering of the hopping barrier.

References

1.	J. Yin, D. Zhao, Y. Qiao, et al. “Bifunctional Spacer Cation Enables High-Efficiency Chiral Red Quasi-2d Perovskite Light-Emitting Diodes Via Defect Passivation and Chirality Enhancement,” *ACS Photonics* 13 (2026): 263-273. <http://doi.org/10.1021/acsphotonics.5c02211>

2.	Y. J. Choi, H. Guo, J. Han, et al. “Structural Distortion-Driven Chirality Transfer and Circularly Polarized Light Emission in Quasi-2D Perovskites Based Light-Emitting Diodes,” *Advanced Functional Materials* (2025): e25181. <http://doi.org/10.1002/adfm.202525181>

3.	L. Jiang, G. Zhang, H. Chen, et al. “Self-Assembled Monolayers Facilitate Simultaneous Enhancements of External Quantum Efficiencies and Circularly Polarized Luminescence Dissymmetry Factors for Chiral Perovskite Red Spin-Light Emitting Diodes,” *Advanced Science* (2025): e12057. <http://doi.org/10.1002/advs.202512057>

4.	M. Mustaqeem, Z. B. Jin, W. C. Tsai, et al. “Mechanically Tuneable Circularly Polarized Flexible Spin Light Emitting Diodes,” *Advanced Optical Materials* 13 (2025): 2500060. <http://doi.org/10.1002/adom.202500060>

5.	J. W. Yao, Y. L. Huang, H. F. Sun, et al. “Efficient Spin-Light-Emitting Diodes with Tunable Red to near-Infrared Emission at Room Temperature,” *Advanced Materials* 37 (2025): 2413669. <http://doi.org/10.1002/adma.202413669>

6.	Q. Q. Wang, H. M. Zhu, Y. Z. Tan, et al. “Spin Quantum Dot Light-Emitting Diodes Enabled by 2d Chiral Perovskite with Spin-Dependent Carrier Transport,” *Advanced Materials* 36 (2024): 2305604. <http://doi.org/10.1002/adma.202305604>

7.	M. Mustaqeem, P.-T. Chou, S. Kamal, et al. “Solution-Processed and Room-Temperature Spin Light-Emitting Diode Based on Quantum Dots/Chiral Metal-Organic Framework Heterostructure,” *Advanced Functional Materials* 33 (2023): 2213587. <http://doi.org/10.1002/adfm.202213587>

8.	C. H. Yang, S. B. Xiao, H. Xiao, L. J. Xu and Z. N. Chen. “Efficient Red-Emissive Circularly Polarized Electroluminescence Enabled by Quasi-2D Perovskite with Chiral Spacer Cation,” *ACS nano* 17 (2023): 7830-7836. <http://doi.org/10.1021/acsnano.3c01054>

9.	T. Duan, J. Ai, S. Chen, et al. “Chiral CdSe/CdS Quantum Dot (in Rod)-Light-Emitting Diodes with Circularly Polarized Electroluminescence,” *Nano Research* 15 (2022): 9573-9577. <http://doi.org/10.1007/s12274-022-4536-7>

10.	Y. H. Kim, Y. X. Zhai, H. P. Lu, et al. “Chiral-Induced Spin Selectivity Enables a Room-Temperature Spin Light-Emitting Diode,” *Science* 371 (2021): 1129. <http://doi.org/10.1126/science.abf5291>

11.	M. H. Lee, Y.-J. Choi, J. Kim, et al. “Circularly Polarized Electroluminescence from Thermally Evaporated Core-Shell Perovskite-Based Light-Emitting Diodes,” *Nano Energy* 151 (2026): 111859. <https://doi.org/10.1016/j.nanoen.2026.111859>

12.	D. Chen, B. Tang, A. A. Sergeev, et al. “Green Spin Light-Emitting Diodes Enabled by Perovskite Nanocrystals in Situ Modified with Chiral Ligands,” *ACS Energy Letters* 10 (2025): 815-821. <http://doi.org/10.1021/acsenergylett.4c03515>

13.	Y. Li, L. Z. Jiang, J. Tang, et al. “Unraveling Chiral Perovskite Spin-Light Emitting Diode Performance and Magneto-Chiroptical Properties Relationship Due to the Synergistic Effect,” *Advanced Functional Materials* 35 (2025): 2424619. <https://doi.org/10.1002/adfm.202424619>

14.	S. He, W. X. Lin, D. Yu, et al. “Perovskite Spin Light-Emitting Diodes with Simultaneously High Electroluminescence Dissymmetry and High External Quantum Efficiency,” *Nature Communications* 16 (2025): 2201. <http://doi.org/10.1038/s41467-025-57472-8>

15.	L. S. Yang, C. Y. Huang, C. A. Hsu, H. Z. Chiu, P. H. Lo and Y. C. Chao. “Room-Temperature Solution-Processed Spin Organic Light-Emitting Diodes Based on Chiral 2D Halide Perovskites,” *Nanoscale* 17 (2025): 17784-17794. <http://doi.org/10.1039/d5nr01117e>

16.	R. Zhang, Y. F. Tian, C. Y. Ye, et al. “Efficient Quasi-2D Perovskite Spin Light-Emitting Diodes Based on Chiral-Induced Spin Selectivity,” *Chemistry of Materials* 36 (2024): 3812-3819. <http://doi.org/10.1021/acs.chemmater.4c00190>

17.	J. Yao, Z. Wang, Y. Huang, et al. “Efficient Green Spin Light-Emitting Diodes Enabled by Ultrafast Energy- and Spin-Funneling in Chiral Perovskites,” *Journal of the American Chemical Society* 146 (2024): 14157-14165. <http://doi.org/10.1021/jacs.4c02821>

18.	H. Gao, Y. Chen, R. Zhang, et al. “Dual-Ligand Quasi-2d Perovskites with Chiral-Induced Spin Selectivity for Room Temperature Spin-LEDs,” *Materials Horizons* 11 (2024): 2906-2913. <http://doi.org/10.1039/D3MH02029K>

19.	Z. Li, J. Wang, S. Chi, K. Lin, W. Zhang and C. He. “Efficient Circularly Polarized Electroluminescence Enabled by Low-Dimensional Bichiral Perovskite Nanocrystals,” *ACS nano* 18 (2024): 34962-34970. <http://doi.org/10.1021/acsnano.4c13260>

20.	J. Tang, S. Tao, Y. Li, et al. “Chiral Ionic Liquids Enable High-Performance Room Temperature Single Junction Spin-Light Emitting Diodes,” *Laser & Photonics Reviews* 19 (2024): 2401008. <https://doi.org/10.1002/lpor.202401008>

21.	B. Li, Y. Li, W. Yuan, et al. “Chiral Quasi-2D Perovskites Based Single Junction Spin-Light-Emitting Diodes,” *Advanced Functional Materials* 35 (2025): 2415433. <https://doi.org/10.1002/adfm.202415433>

22.	L.-S. Yang, C.-Y. Huang, C.-A. Hsu, et al. “Solution-Processed Spin Organic Light-Emitting Diodes Based on Antisolvent-Treated 2d Chiral Perovskites with Strong Spin-Dependent Carrier Transport,” *Materials Horizons* 12 (2025): 1863-1877. <http://doi.org/10.1039/D4MH01371A>

23.	L. Z. Feng, Y. H. Song, Z. D. Li, et al. “Dimensional and Doping Engineering of Chiral Perovskites with Enhanced Spin Selectivity for Green Emissive Spin Light-Emitting Diodes,” *Nano Letters* 24 (2024): 6084-6091. <http://doi.org/10.1021/acs.nanolett.4c01138>

24.	M. P. Hautzinger, X. Pan, S. C. Hayden, et al. “Room-Temperature Spin Injection across a Chiral Perovskite/Iii–V Interface,” *Nature* 631 (2024): 307-312. <http://doi.org/10.1038/s41586-024-07560-4>

25.	G. Jang, D. Y. Jo, S. Ma, et al. “Core-Shell Perovskite Quantum Dots for Highly Selective Room-Temperature Spin Light-Emitting Diodes,” *Advanced Materials* 36 (2024): 2309335. <http://doi.org/10.1002/adma.202309335>

26.	C.-H. Yang, H. Xiao, Y.-F. Sang, et al. “In Situ Formed Perovskite Nanocrystal Films toward Efficient Circularly Polarized Electroluminescence,” *Advanced Functional Materials* 34 (2023): 2310500. <http://doi.org/10.1002/adfm.202310500>

27.	C. Ye, J. Jiang, S. Zou, W. Mi and Y. Xiao. “Core-Shell Three-Dimensional Perovskite Nanocrystals with Chiral-Induced Spin Selectivity for Room-Temperature Spin Light-Emitting Diodes,” *Journal of the American Chemical Society* 144 (2022): 9707-9714. <http://doi.org/10.1021/jacs.2c01214>

28.	X. Yan, R. Cao, R. Zhang, H. Gao and Y. Xiao. “Mixed-Ligand Chiral Quasi-2D Perovskites for Standard and Deep Blue CP-LEDs,” *Advanced Functional Materials* 34 (2024): 2410012. <https://doi.org/10.1002/adfm.202410012>

29.	G. S. Zhang, J. Tang, Y. Li, et al. “High-Performance Sky-Blue Perovskite Spin-Light Emitting Diodes Due to Chiral Ionic Liquid Implantation and Passivation,” *Advanced Functional Materials* 35 (2025): 2503088. <http://doi.org/10.1002/adfm.202503088>

30.	Z. Zheng, Q. Hu, S. Zhang, et al. “A Highly Efficient Non-Fullerene Organic Solar Cell with a Fill Factor over 0.80 Enabled by a Fine-Tuned Hole-Transporting Layer,” *Advanced Materials* 30 (2018): 1801801. <http://doi.org/10.1002/adma.201801801>

31.	A. Khetubol, A. Hassinen, Y. Firdaus, et al. “Study of Hole Mobility in Poly(N-Vinylcarbazole) Films Doped with CdSe/ZnS Quantum Dots Encapsulated by 11-(N-Carbazolyl) Undecanoic Acid (C11),” *Journal of Applied Physics* 114 (2013): 173704. <http://doi.org/10.1063/1.4827395>

32.	H. H. Fong, A. Papadimitratos and G. G. Malliaras. “Nondispersive Hole Transport in a Polyfluorene Copolymer with a Mobility of 0.01cm^2^v^−1^s^−1^,” *Applied Physics Letters* 89 (2006): 172116. <http://doi.org/10.1063/1.2369545>

33.	D. B. C and P. Sriramalakshmi. “Simulation of Lead Free Heterojunction N-Fasni3/P-Frgecl3 Based Perovskite Solar Cell with ZnMgO as Electron Transport Layer and GO as Hole Transport Layer Using Scaps-1D,” *Results in Engineering* 27 (2025): 106180. <https://doi.org/10.1016/j.rineng.2025.106180>

34.	W. F. Pasveer, J. Cottaar, C. Tanase, et al. “Unified Description of Charge-Carrier Mobilities in Disordered Semiconducting Polymers,” *Physical Review Letters* 94 (2005): 206601. <http://doi.org/10.1103/PhysRevLett.94.206601>

35.	R. J. de Vries, S. L. M. van Mensfoort, V. Shabro, S. I. E. Vulto, R. A. J. Janssen and R. Coehoorn. “Analysis of Hole Transport in a Polyfluorene-Based Copolymer— Evidence for the Absence of Correlated Disorder,” *Applied Physics Letters* 94 (2009): 163307. <http://doi.org/10.1063/1.3119317>
